# Vasculitis associated with VEXAS syndrome: A literature review

**DOI:** 10.3389/fmed.2022.983939

**Published:** 2022-08-15

**Authors:** Ryu Watanabe, Manami Kiji, Motomu Hashimoto

**Affiliations:** Department of Clinical Immunology, Osaka Metropolitan University Graduate School of Medicine, Osaka, Japan

**Keywords:** autoinflammatory disease, giant cell arteritis, leukocytoclastic vasculitis, vasculitis, VEXAS syndrome

## Abstract

Vasculitis is an inflammatory disorder of the blood vessels that causes damage to a wide variety of organs through tissue ischemia. Vasculitis is classified according to the size (large, medium, or small) of the blood vessels. In 2020, VEXAS (vacuoles, E1 enzyme, X-linked, autoinflammatory, somatic) syndrome, a novel autoinflammatory syndrome, was described. Somatic mutations in methionine-41 of UBA1, the major E1 enzyme that initiates ubiquitylation, are attributed to this disorder. This new disease entity connects seemingly unrelated conditions: inflammatory syndromes (relapsing chondritis, Sweet's syndrome, or neutrophilic dermatosis) and hematologic disorders (myelodysplastic syndrome or multiple myeloma). Notably, such patients sometimes develop vasculitis, such as giant cell arteritis and polyarteritis nodosa, and fulfill the corresponding classification criteria for vasculitis. Thus, vasculitis can be an initial manifestation of VEXAS syndrome. In this research topic exploring the link between autoinflammatory diseases and vasculitis, we first provide an overview of the disease mechanisms and clinical phenotypes of VEXAS syndrome. Then, a literature review using the PubMed database was performed to delineate the clinical characteristics of vasculitis associated with VEXAS syndrome. Finally, the therapeutic options and unmet needs of VEXAS syndrome are discussed.

## Introduction

In 1997, gain-of-function mutations in *MEFV* were reported to cause familial Mediterranean fever (FMF) (1). Based on this discovery, the concept of autoinflammation was proposed to delineate a group of disorders characterized by recurrent episodes of fever and systemic inflammation (2). Since then, advances in genomic techniques have identified multiple monogenic disorders and their corresponding signaling pathways (3–5). Accordingly, the disease concept of autoinflammation has shifted from monogenic disorders to complex multifactorial conditions (6).

Autoimmunity and autoinflammation were considered distinct disease entities, but it has become clear that they form a spectrum of diseases, with monogenic autoinflammatory diseases and autoimmune diseases characterized by multiple autoantibodies representing the two ends (7, 8). Systemic lupus erythematosus is a representative autoimmune disease that depends on acquired immunity, whereas FMF and tumor necrosis factor (TNF) receptor-associated periodic syndromes are the best described autoinflammatory diseases in which innate immunity considerably contributes to pathogenesis (9, 10). Most rheumatic and musculoskeletal diseases show a mixed pattern of autoinflammation and autoimmunity.

In 2020, a novel autoinflammatory syndrome [vacuoles, E1 enzyme, X-linked, autoinflammatory, and somatic (VEXAS) syndrome] was reported (11). Hereafter, we refer to this paper as “the initial report”. This new disease is attributable to somatic mutations in methionine-41 of UBA1, the major E1 enzyme that initiates ubiquitylation. Surprisingly, this syndrome connects seemingly unrelated conditions, such as inflammatory syndromes (relapsing chondritis, Sweet's disease, or neutrophilic dermatosis) and hematologic disorders (myelodysplastic syndrome or multiple myeloma). Each organ manifestation often fulfills the corresponding diagnostic or classification criteria. Thus, this syndrome is considered to exhibit a mixed pattern of autoinflammation and autoimmunity.

In addition, patients with VEXAS syndrome sometimes develop vasculitis such as giant cell arteritis (GCA) and polyarteritis nodosa (PAN) (11). However, as case reports and series of VEXAS syndrome have been accumulated, various types of coexisting vasculitis have been reported, including leukocytoclastic vasculitis (LCV), immunoglobulin A (IgA) vasculitis, and antineutrophil cytoplasmic antibody (ANCA)-associated vasculitis (AAV) (12–14). Moreover, this disease was considered to be found only in males, since *UBA1* lies on the X chromosome. However, several female cases of inherited or acquired monosomy of the X chromosome have been reported (15–18). Thus, the clinical picture of VEXAS syndrome is heterogeneous and continuously expanding.

In this review, we provide an overview of the disease mechanisms and clinical phenotypes of VEXAS syndrome. We focused on the clinical characteristics of vasculitis associated with VEXAS syndrome through a literature review using the PubMed database. Finally, we discuss the therapeutic options and unmet needs of this syndrome.

## How do somatic mutations in UBA1 cause VEXAS syndrome?

Ubiquitylation is a multi-step post-translational modification that triggers proteasomal degradation (19). In this process, ubiquitin covalently binds to the substrate sequentially. Ubiquitylation is essential for multiple cellular processes such as cell cycle progression, DNA damage response, and immune signaling pathways (20, 21). Thus, dysregulation of the ubiquitin-proteasome system results in many disease states, such as infantile neurodegeneration, susceptibility to infection, lymphoproliferative disorders, and malignancy (21–23). Several autoinflammatory diseases have also been linked to alterations in the ubiquitylation system (24).

Ubiquitylation is initiated by the attachment of a single ubiquitin molecule to a target protein through a three-step enzymatic cascade that includes ubiquitin-activating (E1), ubiquitin-conjugating (E2), and ubiquitin-ligating (E3) enzymes (25). The concerted action of E1, E2, and E3 enzymes, as well as deubiquitylases, generates specific ubiquitylation patterns, which trigger the recognition and degradation of substrates by the proteasome. Ubiquitin-like modifier-activating enzyme 1 (UBA1), the major E1 enzyme, has two isoforms: UBA1a and UBA1b (26). UBA1a is the long isoform starting from codon 1 (Met1) of UBA1 and is localized in the nucleus, whereas UBA1b is the short isoform starting from codon 41 (Met41) and is localized in the cytoplasm without a nuclear localization signal.

Individuals with VEXAS syndrome acquire missense mutations at or around the start codon for UBA1b (Met41), which abrogates the expression of UBA1b. More than half of myeloid and erythroid precursor cells harbor such loss-of-function mutations (11). Therefore, somatic mutations found in VEXAS syndrome lead to a reduction in cytoplasmic UBA1 function, and the resultant decreased ubiquitylation activates the unfolded protein response and type I interferon production (21). Indeed, transcriptome analysis of the peripheral blood from patients with VEXAS syndrome showed highly activated inflammatory signatures in multiple pathways, including TNF, interleukin-6 (IL-6), interferon-γ, and IL-8 (11). Neutrophils may also participate in exacerbating the inflammatory response by enhancing neutrophil extracellular trap (NET) formation (27).

## Clinical phenotypes of VEXAS syndrome

### Chondritis

**Figure 1:**
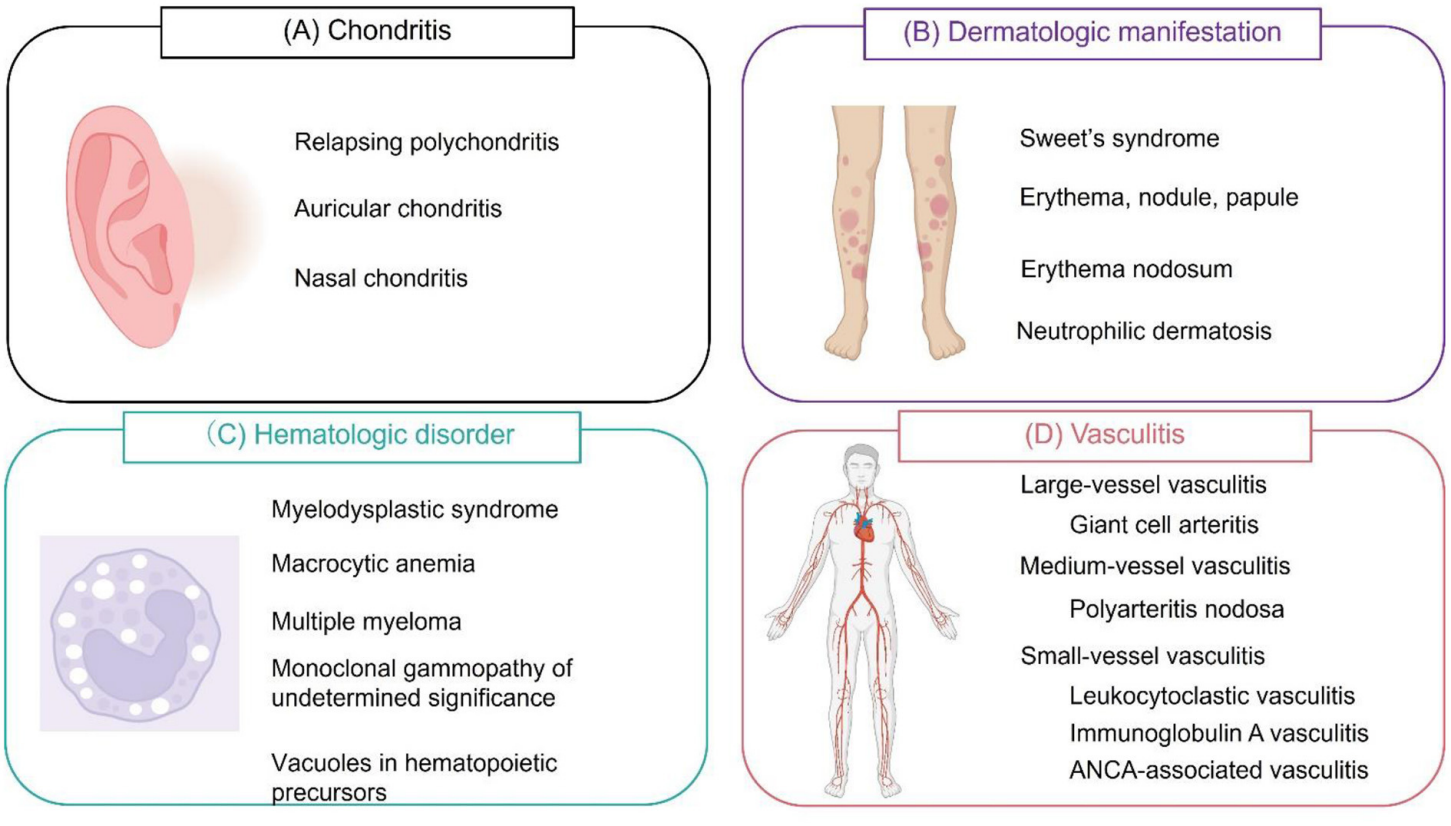
Symptoms of VEXAS syndrome. **(A)** Relapsing polychondritis (auricular and/or nasal chondritis) is one of the most common manifestations of VEXAS (vacuoles, E1 enzyme, X-linked, autoinflammatory, somatic) syndrome. **(B)** Dermatological manifestations include Sweet's syndrome, erythema, nodules, papules, erythema nodosum, and panniculitis. These lesions are histologically characterized by neutrophilic dermatosis. **(C)** Hematological manifestations include myelodysplastic syndrome, macrocytic anemia, multiple myeloma, and monoclonal gammopathy of undetermined significance. Vacuole formation is characteristic of hematopoietic and myeloid precursor cells. **(D)** VEXAS syndrome can cause inflammation of blood vessels of any size. Cranial or extracranial giant cell arteritis, polyarteritis nodosa, leukocytoclastic vasculitis, immunoglobulin A vasculitis, and antineutrophil cytoplasmic antibody (ANCA)-associated vasculitis can be observed. Leukocytoclastic vasculitis is the most common type of vasculitis.

In the initial report, 25 patients were diagnosed with VEXAS syndrome based on the confirmation of somatic mutations in codon 41 of *UBA1* (p.Met41Val, p.Met41Thr, or p.Met41Leu) (11). The median age at disease onset was 64 years, and all patients were male. Auricular and/or nasal chondritis was one of the most common organ manifestations (Figure 1), with 15 (60%) patients meeting the classification criteria for relapsing polychondritis (RP). According to other cohort studies, the incidence of chondritis was 36–50% (28–30).

The frequency of *UBA1* mutations in patients with RP has varied greatly in previous reports, ranging from 7.6% (7/92 patients) to 72.7% (8/11 patients) (17, 31). The reason for this difference remains unclear but could be due to racial differences. RP patients with *UBA1* mutations (VEXAS-RP) had significantly higher mortality rates than those without, necessitating the early identification of VEXAS-RP (31). A decision tree algorithm based on male sex, mean corpuscular volume >100 fl, and platelet count <200 ×10^3^/μl may help differentiate VEXAS-RP from RP (31).

### Skin manifestations

Skin manifestations are also common in VEXAS syndrome, including Sweet's syndrome, erythema, nodules, papules, erythema nodosum, and panniculitis (14, 29, 32–36). The histological hallmark of skin lesions is neutrophilic dermatosis, which is often accompanied by LCV. The most commonly affected sites are the neck and trunk, but they also appear in the extremities (37). According to a literature review, approximately 90% (126/141) of published cases of VEXAS syndrome had cutaneous signs (14).

Zakine et al. performed molecular analyses of skin tissue samples and demonstrated that dermal infiltrates are derived from pathological myeloid clones with *UBA1* mutations (34). However, Lacombe et al. identified *UBA1* mutations only in neutrophilic dermatosis, but not in non-neutrophilic dermatosis, suggesting a distinction between “clonal” (neutrophilic dermatosis) and “paraclonal” (LCV and panniculitis) cutaneous involvements (37).

### Hematologic disorder

Hematologic disorders are also prevalent in VEXAS syndrome, including myelodysplastic syndrome (MDS), macrocytic anemia, multiple myeloma, and monoclonal gammopathy of undetermined significance (38). The name “V”EXAS syndrome stems from “vacuoles” in myeloid and erythroid cells, but not in T lymphocytes, B lymphocytes, or fibroblasts (11). Of note, myeloid and erythroid vacuolization is not specific to VEXAS syndrome, but has been described in other disorders, including copper deficiency (39), acute myeloid leukemia (40), Menkes disease (41), and transcobalamin II deficiency (42).

Thrombotic manifestations occur in approximately 40% of patients with VEXAS syndrome (43). The reported incidence of venous thromboembolism (36.4%) is much higher than that of arterial thrombosis (1.6%) (44). Crosstalk between aberrantly activated immune cells, platelets, and endothelial cells may result in the dysregulation of hemostasis and endothelial dysfunction.

### Other clinical manifestations

Lung involvement, such as pulmonary infiltration, organizing pneumonia, and pleural effusion, can be found in approximately 50–70% of patients with VEXAS syndrome (11, 29, 30). Other symptoms include recurrent fever, arthritis, lymphadenopathy, and ocular manifestations. A wide array of clinical manifestations necessitate generalists, primary care providers, and rheumatologists to familiarize themselves with this syndrome (45).

## Vasculitis associated with VEXAS syndrome

### Large vessel vasculitis

In the initial report, 1 of 25 patients (4%) was diagnosed with GCA based on temporal artery biopsy. A 77-year-old male with a *UBA1* mutation (p.Met41Val) presented with fever, pulmonary infiltrates, deep vein thrombosis, and MDS. Despite treatment with glucocorticoids, the patient died at 78 years old (11). As of June 25, 2022, 102 papers regarding VEXAS syndrome have been published in the PubMed database when the search term “VEXAS” was applied, and all papers were carefully reviewed. Table 1 summarizes the vasculitides associated with VEXAS syndrome. To the best of our knowledge, another case was diagnosed as extra-cranial GCA (46). This patient was a male in his sixties presenting with fever, pulmonary infiltrates, ear and nose chondritis, macrocytic anemia, and thrombocytopenia with a *UBA1* mutation (p.Met41Thr). 18F-fluorodeoxyglucose positron emission tomography showed increased uptake in the aortic wall of the ascending aorta and aortic arch. The patient showed resistance to glucocorticoids and multiple biological disease-modifying antirheumatic drugs (bDMARDs) such as tocilizumab, anakinra, and infliximab, and died 8 years later (Table 1). Since only two cases of VEXAS-GCA have been reported, sufficient examination to discriminate GCA and VEXAS-GCA could not be conducted. The differential diagnoses of large vessel vasculitis are diverse (53), and these cases illustrate that VEXAS syndrome could be a potential mimicker of large vessel vasculitis (54).

**Table 1:** Vasculitis associated with VEXAS syndrome identified by our literature review.

	**Case**	**Age at onset**	**Sex**	**Vasculitis**	**UBA1 mutation**	**Chondritis**	**Skin**	**MDS**	**Macrocytic anemia**	**Pulmonary infiltrates**	**Use of GCs**	**Use of b/tsDMARDs**	**Prognosis**	**References**
Large vessel vasculitis	1	77	M	GCA	p.Met41Val	–	–	+	+	+	+	–	deceased	(11)
	2	60 s	M	GCA	p.Met41Thr	+	–	–	+	+	+	TCZ, ANK, IFX	deceased	(46)
Medium vessel vasculitis	1	56	M	PAN	p.Met41Val	–	+	+	+	+	+	IFX, ANK	deceased	(11)
	2	55	M	PAN	p.Met41Val	+	+	+	+	+	+	ANK, CAN, ADA, IFX	deceased	(11)
	3	80	M	PAN	p.Met41Thr	–	+	+	+	+	+	–	deceased	(11)
	4	80	M	MVV	+	–	+	–	+	+	+	–	deceased	(35)
	5	50	M	PAN	p.Met41Thr	–	+	–	+	pulmonary nodule	+	IFX, RTX	deceased	(47)
	6	74	M	PAN	p.Met41Val	–	+	–	+	pulmonary infarction	+	RTX, TCZ	Survived	(48)
	7	43	M	PAN	p.Met41Val	–	+	–	+	–	+	ANK, CAN	Survived	(49)
	8	63	M	PAN	p.Met41Leu	+	+	–	+	–	+	ANK, CAN, TCZ	Survived	(49)
	9	55	M	MVV	p.Met41Val	–	+	–	+	–	NA	NA	deceased	(50)
Small vessel vasculitis	1	72	M	LCV	Not tested	–	+	+	+	+	+	–	NA	(12)
	2	55	M	LCV	p.Met41Val	–	+	+	+	+	+	TCZ, CAN, ETN	NA	(51)
	3	68	M	LCV	p.Met41Thr	–	+	+	+	+	+	TCZ, ADA, CAN	waiting HSCT	(52)
	4	72	M	LCV	p.Met41Thr	NA	NA	NA	NA	NA	NA	NA	NA	(37)
	5	63	M	LCV	p.Met41Leu	NA	NA	NA	NA	NA	NA	NA	NA	(37)
	6	87	M	LCV	p.Met41Val	NA	NA	NA	NA	NA	NA	NA	NA	(37)
	7	55	M	LCV	p.Met41Val	+	+	+	+	+	+	ANK, RTX	Survived	(30)
	8	69	M	LCV	p.Met41Thr	+	+	+	+	–	+	ANK, TCZ	Survived	(30)
	9	74	M	LCV	p.Met41Thr	+	+	–	+	–	+	–	Survived	(30)
	10	76	M	IgAV	p.Met41Thr	+	+	+	+	+	+	TCZ	Survived	(13)
	11	72	M	MPA	p.Met41Val	–	–	+	+	+	+	RTX	Survived	(12)
	12	71	M	GPA	+	–	+	–	anemia	–	+	IFX, RTX, TCZ	Survived	(35)

ANK, anakinra; bDMARDs, biological disease-modifying antirheumatic drugs; CAN, canakinumab; ETN, etanercept; GC, glucocorticoid; GCA, giant cell arteritis; GPA, granulomatosis with polyangiitis; HSCT, hematopoietic stem cell transplantation; IFX, infliximab; IgAV, IgA vasculitis; LCV, leukocytoclastic vasculitis; MDS, myelodysplastic syndrome; MPA, microscopic polyangiitis; MVV, medium vessel vasculitis; NA, not available; PAN, polyarteritis nodosa; RTX, rituximab; TCZ, tocilizumab; VEXAS, vacuoles, E1 enzyme, X-linked, autoinflammatory, somatic.

The incidence of GCA in reported cases with VEXAS syndrome seems rare. The question is how often *UBA1* mutations are found in patients with GCA. To address this, Poulter et al. sequenced *UBA1* in 612 male samples obtained from the UK GCA Consortium. No samples showed *UBA1* mutations, whereas the mutation was identified in seven out of 1,055 samples from the cytopenic cohorts (55). The authors concluded that VEXAS syndrome is rarely misdiagnosed as GCA in the UK. However, as the incidence of GCA varies greatly by race, perhaps due to HLA differences (56, 57), race-specific *UBA1* mutations in patients with GCA need to be assessed.

### Medium vessel vasculitis

In the initial report, 3 (12%) patients with VEXAS syndrome fulfilled the classification criteria for PAN (58), which is a medium-sized vessel vasculitis (59). Our literature review found nine cases of medium vessel vasculitis (Table 1) (11, 35, 47–50). The average age at disease onset was 61.8 years (ranging from 43 to 80 years) and all were male. Seven of the nine patients fulfilled the classification criteria for PAN. Macrocytic anemia and skin lesions were observed in all cases, while chondritis was found in only two of the nine cases. Despite the use of high-dose glucocorticoids and multiple bDMARDs, such as infliximab, anakinra, and rituximab, six of the nine patients died during the treatment course. Notably, two of the three survivors successfully underwent allogeneic hematopoietic stem cell transplantation (49).

### Small vessel vasculitis

The most common vasculitis is small-vessel vasculitis, particularly LCV. The reason stems from the fact that skin manifestations, which are observed in 80–90% of patients, often reveal the histopathology of LCV (14, 37, 60). LCV is histologically characterized by angiocentric segmental inflammation, fibrinoid necrosis, and neutrophilic infiltration around the blood vessel walls (14, 61). In the initial report, LCV was histologically confirmed in seven of 25 patients (28%) with VEXAS syndrome and in seven of 22 patients (31.8%) with dermatologic manifestations. Although the demographics of patients who developed LCV was unclear in the initial report, an additional nine cases of LCV were identified in our literature review (Table 1) (12, 30, 37, 51, 52). The average age at disease onset was 68.3 years (ranging from 55 to 87 years) and all were male. Clinical features of these patients seem not different from patients without LCV.

Furthermore, it has become clear that VEXAS syndrome can be associated with small vessel vasculitides other than LCV, such as IgA vasculitis (13) and AAV (microscopic polyangiitis and granulomatosis with polyangiitis) (12, 35). It remains unclear whether the complications of these diseases are coincidental or causative; however, aberrant neutrophil activation with excessive NETs formation is a common feature of both VEXAS syndrome and AAV (27, 62). In AAV, ANCA activates neutrophils to produce reactive oxygen species and extrude chromosomal DNA in the form of NETs. The neutrophil activation highly depends on the priming by TNF, lipopolysaccharide, or complement factor 5a (C5a) (62). The efficacy and safety of C5 receptor blockade by avacopan has been demonstrated in patients with AAV (63), but has not yet been tested in VEXAS syndrome.

## Current therapeutic approach for VEXAS syndrome

### Immunosuppressive agents and DMARDs

Considering the inflammatory aspect, immunosuppressive therapies are a sensible treatment option for this syndrome. Glucocorticoids, cyclophosphamide, conventional synthetic DMARDs (methotrexate, mycophenolate mofetil, azathioprine), and bDMARDs [including anti-IL-1 (anakinra, canakinumab), anti-TNF (infliximab and adalimumab), anti-IL-6 (tocilizumab), anti-CD20 (rituximab), anti-IL-17 (secukinumab), anti-IL-12/IL-23 (ustekinumab) therapies, and abatacept] have been administered to patients with VEXAS syndrome (11, 47, 64–66). In addition, janus kinase (JAK) inhibitors, such as ruxolitinib, tofacitinib, and baricitinib, have been introduced to some patients to block intracellular cytokine signalings (67–70). Given that multiple cytokines are involved in the disease mechanism of VEXAS syndrome, use of JAK inhibitors seems better therapeutic strategy rather than single cytokine blockade. However, these therapies work great for some patients and not for others, yielding varying results. Biomarkers that distinguish patients who are responsive to these therapies and those who are not should be established.

### Hypomethylating agents (azacytidine)

Since abnormal DNA methylation patterns drive the disease mechanisms of MDS, standard therapies for MDS include hypomethylating agents such as azacytidine (71). Considering the high prevalence of MDS in VEXAS syndrome, azacytidine could be a good candidate for this syndrome (67). Indeed, there have been a case report of successful treatment with azacytidine (72). However, in the French registry, 11 patients with VEXAS syndrome and MDS were treated with azacytidine, and the clinical response was achieved only in five patients (73), suggesting that this agent does not necessarily guarantee sufficient therapeutic effect and long-term prognosis.

### Allogeneic hematopoietic stem cell transplantation

If above-mentioned therapies fail, allogeneic hematopoietic stem cell transplantation (ASCT) may be the last treatment option for VEXAS syndrome. Although the incidence of treatment-related mortality remains high, given that irreversible somatic mutations have already been introduced in myeloid precursor cells in VEXAS syndrome, rejuvenation of the immune system by ASCT may be an ideal treatment. In fact, a series of cases showing the successful treatment courses with ASCT has been accumulated (49, 74).

## Unmet needs in VEXAS syndrome

### Diagnosis

VEXAS syndrome was discovered using an innovative genotype-driven approach. Currently, the diagnosis of this syndrome depends solely on the presence of *UBA1* mutations confirmed by Sanger sequencing, the gold standard for genetic sequencing (75). However, Sanger sequencing method is time-consuming and has several limitations such as the missed recognition of variations (76). Therefore, it remains unclear whether this is sufficient. Use of next-generation sequencing (NGS), including whole-genome sequencing and whole-exome sequencing, may favorably serve in the faster and more accurate diagnosis.

In addition, classification criteria for the syndrome should be established to conduct clinical studies. In addition to genetic sequencing, a point-based system that weighs each clinical manifestation may be required.

### Treatment

As described, VEXAS syndrome shows resistance to multiple therapeutic agents, resulting in high mortality rates. Early identification of this syndrome by NGS and classification criteria may alter the treatment course. Furthermore, if the precise mechanism by which somatic mutations in *UBA1* occur in middle-aged or elderly males is elucidated, a more disease-specific therapeutic approach for this syndrome, and even prevention of this mutation, could be possible.

In conclusion, VEXAS syndrome has led to the recognition that somatic mutations may be a more frequent cause of human disease than previously recognized (77). Further studies are required to provide appropriate diagnosis and treatment.

## Author contributions

MK and RW conducted a literature review and generated Figure and Table. RW drafted the manuscript. MH finalized the manuscript. All authors contributed to the article and approved the submitted version.

## Funding

This work was in part supported by JSPS KAKENHI Grant Numbers 20K17418 and 22K08569, a grant-in-aid of the Cardiovascular Research Fund, Tokyo, Japan, and a Grant for Promoting Research and Survey in Rheumatic Diseases by Japan Rheumatism Foundation to RW.

## Conflict of interest

Author RW receives speaker's fee from Eli Lilly. Author MH receives research grants from AbbVie, Asahi-Kasei, Brystol-Meyers, Eisai, Eli Lilly, and Novartis Pharma. The remaining author declares that the research was conducted in the absence of any commercial or financial relationships that could be construed as a potential conflict of interest.

## Publisher's note

All claims expressed in this article are solely those of the authors and do not necessarily represent those of their affiliated organizations, or those of the publisher, the editors and the reviewers. Any product that may be evaluated in this article, or claim that may be made by its manufacturer, is not guaranteed or endorsed by the publisher.

## References

[1] Bernot A Clepet C Dasilva C Devaud C Petit JL Caloustian C. A candidate gene for familial Mediterranean fever. Nat Genet. (1997) 17:25–31. 10.1038/ng0997-25 9288094

[2] McDermott MF Aksentijevich I Galon J McDermott EM Ogunkolade BW Centola M. Germline mutations in the extracellular domains of the 55 kDa TNF receptor, TNFR1, define a family of dominantly inherited autoinflammatory syndromes. Cell. (1999) 97:133–44. 10.1016/S0092-8674(00)80721-7 10199409

[3] Kastner DL Aksentijevich I Goldbach-Mansky R. Autoinflammatory disease reloaded: a clinical perspective. Cell. (2010) 140:784–90. 10.1016/j.cell.2010.03.002 20303869 PMC3541025

[4] Manthiram K Zhou Q Aksentijevich I Kastner DL. The monogenic autoinflammatory diseases define new pathways in human innate immunity and inflammation. Nat Immunol. (2017) 18:832–42. 10.1038/ni.3777 28722725

[5] Stone DL Beck DB Manthiram K Park YH Chae JJ Remmers E. The systemic autoinflammatory diseases: coming of age with the human genome. J Allergy Clin Immunol. (2020) 146:997–1001. 10.1016/j.jaci.2020.09.014 32987090 PMC11008603

[6] Nigrovic PA Lee PY Hoffman HM. Monogenic autoinflammatory disorders: conceptual overview, phenotype, and clinical approach. J Allergy Clin Immunol. (2020) 146:925–37. 10.1016/j.jaci.2020.08.017 33160483 PMC7727443

[7] Savic S Caseley EA McDermott MF. Moving towards a systems-based classification of innate immune-mediated diseases. Nat Rev Rheumatol. (2020) 16:222–37. 10.1038/s41584-020-0377-5 32107482

[8] Szekanecz Z McInnes IB Schett G Szamosi S Benko S Szucs G. Autoinflammation and autoimmunity across rheumatic and musculoskeletal diseases. Nat Rev Rheumatol. (2021) 17:585–95. 10.1038/s41584-021-00652-9 34341562

[9] Koga T Kawakami A. Diagnosis and treatment of autoinflammatory diseases in adults: a clinical approach from rheumatologists. Immunol Med. (2018) 41:177–80. 10.1080/25785826.2018.1524105 30714492

[10] Migita K Asano T Sato S Koga T Fujita Y Kawakami A. Familial Mediterranean fever: overview of pathogenesis, clinical features and management. Immunol Med. (2018) 41:55–61. 10.1080/13497413.2018.1481579 30938266

[11] Beck DB Ferrada MA Sikora KA Ombrello AK Collins JC Pei W. Somatic mutations in UBA1 and severe adult-onset autoinflammatory disease. N Engl J Med. (2020) 383:2628–38. 10.1056/NEJMoa2026834 33108101 PMC7847551

[12] Muratore F Marvisi C Castrignanò P Nicoli D Farnetti E Bonanno O. VEXAS syndrome: a case series from a single-center cohort of Italian patients with vasculitis. Arthritis Rheumatol. (2022) 74:665–70. 10.1002/art.41992 34611997 PMC8957507

[13] Pàmies A Ferràs P Bellaubí-Pallarés N Giménez T Raventós A Colobran R. VEXAS syndrome: relapsing polychondritis and myelodysplastic syndrome with associated immunoglobulin A vasculitis. Rheumatology. (2022) 61:e69–71. 10.1093/rheumatology/keab782 34668539

[14] Sterling D Duncan ME Philippidou M Salisbury JR Kulasekararaj AG Basu TN. VEXAS syndrome (vacuoles, E1 enzyme, X-linked, autoinflammatory, somatic) for the dermatologist. J Am Acad Dermatol. (2022) S0190-9622(22)00181–5. 10.1016/j.jaad.2022.01.042 35121074

[15] Arlet JB Terrier B Kosmider O. Mutant UBA1 and severe adult-onset autoinflammatory disease. N Engl J Med. (2021) 384:2163. 10.1056/NEJMc2102124 34077651

[16] Barba T Jamilloux Y Durel CA Bourbon E Mestrallet F Sujobert P. VEXAS syndrome in a woman. Rheumatology. (2021) 60:e402–3. 10.1093/rheumatology/keab392 33930131

[17] Tsuchida N Kunishita Y Uchiyama Y Kirino Y Enaka M Yamaguchi Y. Pathogenic UBA1 variants associated with VEXAS syndrome in Japanese patients with relapsing polychondritis. Ann Rheum Dis. (2021) 80:1057–61. 10.1136/annrheumdis-2021-220089 33789873

[18] Stubbins RJ McGinnis E Johal B Chen LY Wilson L Cardona DO. VEXAS syndrome in a female patient with constitutional 45, X (Turner syndrome). Haematologica. (2022) 107:1011–3. 10.3324/haematol.2021.280238 34911285 PMC8968888

[19] Komander D Rape M. The ubiquitin code. Annu Rev Biochem. (2012) 81:203–29. 10.1146/annurev-biochem-060310-170328 22524316

[20] Craney A Rape M. Dynamic regulation of ubiquitin-dependent cell cycle control. Curr Opin Cell Biol. (2013) 25:704–10. 10.1016/j.ceb.2013.07.004 23890701

[21] Beck DB Werner A Kastner DL Aksentijevich I. Disorders of ubiquitylation: unchained inflammation. Nat Rev Rheumatol. (2022) 18:435–47. 10.1038/s41584-022-00778-4 35523963 PMC9075716

[22] Oh E Akopian D Rape M. Principles of ubiquitin-dependent signaling. Annu Rev Cell Dev Biol. (2018) 34:137–62. 10.1146/annurev-cellbio-100617-062802 30110556

[23] Rape M. Ubiquitylation at the crossroads of development and disease. Nat Rev Mol Cell Biol. (2018) 19:59–70. 10.1038/nrm.2017.83 28928488

[24] Aksentijevich I Zhou Q. NF-kappaB pathway in autoinflammatory diseases: dysregulation of protein modifications by ubiquitin defines a new category of autoinflammatory diseases. Front Immunol. (2017) 8:399. 10.3389/fimmu.2017.00399 28469620 PMC5395695

[25] Morreale FE Walden H. Types of ubiquitin ligases. Cell. (2016) 165:248–248. 10.1016/j.cell.2016.03.003 27015313

[26] Schulman BA Harper JW. Ubiquitin-like protein activation by E1 enzymes: the apex for downstream signalling pathways. Nat Rev Mol Cell Biol. (2009) 10:319–31. 10.1038/nrm2673 19352404 PMC2712597

[27] Lacombe V Prevost M Bouvier A Thépot S Chabrun F Kosmider O. Vacuoles in neutrophil precursors in VEXAS syndrome: diagnostic performances and threshold. Br J Haematol. (2021) 195:286–9. 10.1111/bjh.17679 34340250

[28] Koster MJ Kourelis T Reichard KK Kermani TA Beck DB Cardona DO. Clinical heterogeneity of the VEXAS syndrome: a case series. Mayo Clin Proc. (2021) 96:2653–9. 10.1016/j.mayocp.2021.06.006 34489099

[29] Georgin-Lavialle S Terrier B Guedon AF Heiblig M Comont T Lazaro E. Further characterization of clinical and laboratory features in VEXAS syndrome: large-scale analysis of a multicentre case series of 116 French patients. Br J Dermatol. (2022) 186:564–74. 10.1111/bjd.20805 34632574

[30] van der Made CI Potjewijd J Hoogstins A Willems HP Kwakernaak AJ de Sevaux RG. Adult-onset autoinflammation caused by somatic mutations in UBA1: A Dutch case series of patients with VEXAS. J Allergy Clin Immunol. (2022) 149:432–9. 10.1016/j.jaci.2021.05.014 34048852

[31] Ferrada MA Sikora KA Luo Y Wells KV Patel B Groarke EM. Somatic mutations in UBA1 define a distinct subset of relapsing polychondritis patients with VEXAS. Arthritis Rheumatol. (2021) 73:1886–95. 10.1002/art.41743 33779074 PMC13539413

[32] Alhomida F Beck DB George TI Shaffer A Lebiedz-Odrobina D Kovacsovics T. Vacuoles, E1 enzyme, X-linked, autoinflammatory, somatic (VEXAS) syndrome-clinical presentation of a newly described somatic, autoinflammatory syndrome. JAAD Case Rep. (2021) 14:111–3. 10.1016/j.jdcr.2021.06.010 34337120 PMC8313797

[33] Dehghan N Marcon KM Sedlic T Beck DB Dutz JP Chen LYC. Vacuoles, E1 enzyme, X-linked, autoinflammatory, somatic (VEXAS) syndrome: fevers, myalgia, arthralgia, auricular chondritis, and erythema nodosum. Lancet. (2021) 398:621. 10.1016/S0140-6736(21)01430-6 34391501

[34] Zakine E Schell B Battistella M Vignon-Pennamen MD Chasset F Mahévas T. UBA1 variations in neutrophilic dermatosis skin lesions of patients with VEXAS syndrome. JAMA Dermatol. (2021) 157:1349–54. 10.1001/jamadermatol.2021.3344 34495287 PMC8427502

[35] Afsahi V Christensen RE Alam M. VEXAS syndrome in dermatology. Arch Dermatol Res. (2022). 10.1007/s00403-022-02340-4. [Epub ahead of print]. 35201420

[36] Khosravi-Hafshejani T O'Connor M To F Sreenivasan G Shojania K Au S. The spectrum of skin disease in VEXAS syndrome: a report of a novel clinico-histopathologic presentation. J Eur Acad Dermatol Venereol. (2022) 36:e435–7. 10.1111/jdv.17924 35028985

[37] Lacombe V Beucher A Urbanski G Le Corre Y Cottin L Croué A. Distinction between clonal and paraclonal cutaneous involvements in VEXAS syndrome. Exp Hematol Oncol. (2022) 11:6. 10.1186/s40164-022-00262-5 35172893 PMC8848791

[38] Grayson PC Patel BA Young NS. VEXAS syndrome. Blood. (2021) 137:3591–4. 10.1182/blood.2021011455 33971000 PMC8462403

[39] Lazarchick J. Update on anemia and neutropenia in copper deficiency. Curr Opin Hematol. (2012) 19:58–60. 10.1097/MOH.0b013e32834da9d2 22080848

[40] Gajendra S Gupta R Sharma A Gupta R Gogia A. Acute myeloid leukaemia with Pseudo-Chediak-Higashi granules and intracytoplasmic vacuoles. Eur J Haematol. (2011). 10.1111/j.1600-0609.2011.01668.x 21679254

[41] Sayin S Unal S Cetin M Gumruk F. Vacuolization in myeloid and erythroid precursors in a child with menkes disease. Turk J Haematol. (2019) 36:203–4. 10.4274/tjh.galenos.2018.2018.0104 29716882 PMC6682784

[42] Unal S Karahan F Arikoglu T Akar A Kuyucu S. Different presentations of patients with transcobalamin II deficiency: a single-center experience from Turkey. Turk J Haematol. (2019) 36:37–42. 10.4274/tjh.galenos.2018.2018.0230 30185401 PMC6373502

[43] Groarke EM Dulau-Florea AE Kanthi Y. Thrombotic manifestations of VEXAS syndrome. Semin Hematol. (2021) 58:230–8. 10.1053/j.seminhematol.2021.10.006 34802545

[44] Oo TM Koay JTJ Lee SF Lee SMS Lim XR Fan BE. Thrombosis in VEXAS syndrome. J Thromb Thrombolysis. (2022) 53:965–70. 10.1007/s11239-021-02608-y 34817788 PMC8612112

[45] Koster MJ Warrington KJ. VEXAS within the spectrum of rheumatologic disease. Semin Hematol. (2021) 58:218–25. 10.1053/j.seminhematol.2021.10.002 34802543

[46] Midtvedt Ø Stray-Pedersen A Andersson H Gunnarsson R Tveten K Ali MM. A man in his sixties with chondritis and bone marrow failure. Tidsskr Nor Laegeforen. (2022) 142. 10.4045/tidsskr.21.0370 35239266

[47] Kao RL Jacobsen AA Billington CJ Jr Yohe SL Beckman AK Vercellotti GM. A case of VEXAS syndrome associated with EBV-associated hemophagocytic lymphohistiocytosis. Blood Cells Mol Dis. (2022) 93:102636. 10.1016/j.bcmd.2021.102636 34864445

[48] Itagane M Teruya H Kato T Tsuchida N Maeda A Uchiyama Y. VEXAS syndrome presenting as treatment-refractory *polyarteritis nodosa*. Arthritis Rheumatol. (2022) 371:1653. 10.1002/art.42257. [Epub ahead of print]. 35696330

[49] Diarra A Duployez N Fournier E Preudhomme C Coiteux V Magro L. Successful allogeneic hematopoietic stem cell transplantation in patients with VEXAS syndrome: a 2-center experience. Blood Adv. (2022) 6:998–1003. 10.1182/bloodadvances.2021004749 34714914 PMC8945317

[50] Obiorah IE Patel BA Groarke EM Wang W Trick M Ombrello AK. Benign and malignant hematologic manifestations in patients with VEXAS syndrome due to somatic mutations in UBA1. Blood Adv. (2021) 5:3203–15. 10.1182/bloodadvances.2021004976 34427584 PMC8405186

[51] Takahashi N Takeichi T Nishida T Sato J Takahashi Y Yamamura M. Extensive multiple organ involvement in VEXAS syndrome. Arthritis Rheumatol. (2021) 73:1896–7. 10.1002/art.41775 33881233

[52] Lötscher F Seitz L Simeunovic H Sarbu AC Porret NA Feldmeyer L. Case report: genetic double strike: VEXAS and TET2-positive myelodysplastic syndrome in a patient with long-standing refractory autoinflammatory disease. Front Immunol. (2021) 12:800149. 10.3389/fimmu.2021.800149 35126364 PMC8811255

[53] Watanabe R Berry GJ Liang DH Goronzy JJ Weyand CM. Pathogenesis of giant cell arteritis and takayasu arteritis-similarities and differences. Curr Rheumatol Rep. (2020) 22:68. 10.1007/s11926-020-00948-x 32845392 PMC9112376

[54] Ramon A Greigert H Ornetti P Bonnotte B Samson M. Mimickers of large vessel giant cell arteritis. J Clin Med. (2022) 11. 10.3390/jcm11030495. [Epub ahead of print]. 35159949 PMC8837104

[55] Poulter J Consortium UV Morgan A Cargo C Savic S. A high-throughput amplicon screen for somatic UBA1 variants in cytopenic and giant cell arteritis cohorts. J Clin Immunol. (2022). 10.1101/2021.12.08.21266919 35366150

[56] Weyand CM Goronzy JJ. Giant-cell arteritis and polymyalgia rheumatica. N Engl J Med. (2014) 371:1653. 10.1056/NEJMc1409206 25337759

[57] Watanabe R Berry GJ Liang DH Goronzy JJ Weyand CM. Cellular signaling pathways in medium and large vessel vasculitis. Front Immunol. (2020) 11:587089. 10.3389/fimmu.2020.587089 33072134 PMC7544845

[58] Lightfoot RW Jr Michel BA Bloch DA Hunder GG Zvaifler NJ McShane DJ. The American College of Rheumatology 1990 criteria for the classification of polyarteritis nodosa. Arthritis Rheum. (1990) 33:1088–93. 10.1002/art.1780330805 1975174

[59] Jennette JC Falk RJ Bacon PA Basu N Cid MC Ferrario F. 2012 revised international Chapel Hill consensus conference nomenclature of vasculitides. Arthritis Rheum. (2013) 65;1–11. 10.1002/art.37715 23045170

[60] Stubbins RJ Cherniawsky H Chen LYC Nevill TJ. Innovations in genomics for undiagnosed diseases: vacuoles, E1 enzyme, X-linked, autoinflammatory, somatic (VEXAS) syndrome. CMAJ. (2022) 194:E524–e527. 10.1503/cmaj.211770 35410861 PMC9001005

[61] Iglesias-Gamarra A Restrepo JF Matteson EL. Small-vessel vasculitis. Curr Rheumatol Rep. (2007) 9:304–11. 10.1007/s11926-007-0049-3 17688840

[62] Michailidou D Mustelin T Lood C. Role of Neutrophils in systemic vasculitides. Front Immunol. (2020) 11:619705. 10.3389/fimmu.2020.619705 33391289 PMC7774018

[63] Jayne DRW Merkel PA Schall TJ Bekker P Group AS. Avacopan for the treatment of ANCA-associated vasculitis. N Engl J Med. (2021) 384:599–609. 10.1056/NEJMoa2023386 33596356

[64] Kirino Y Takase-Minegishi K Tsuchida N Hirahara L Kunishita Y Yoshimi R. Tocilizumab in VEXAS relapsing polychondritis: a single-center pilot study in Japan. Ann Rheum Dis. (2021) 80:1501–2. 10.1136/annrheumdis-2021-220876 34260381

[65] Magnol M Couvaras L Degboé Y Delabesse E Bulai-Livideanu C Ruyssen-Witrand A. VEXAS syndrome in a patient with previous spondyloarthritis with a favourable response to intravenous immunoglobulin and anti-IL17 therapy. Rheumatology. (2021) 60:e314–5. 10.1093/rheumatology/keab211 33693498

[66] Pathmanathan K Taylor E Balendra J Lim A Carroll G. VEXAS syndrome: favourable clinical and partial haematological responses to subcutaneous abatacept therapy with 30-month follow up. Rheumatology. (2022) 61:e174–7. 10.1093/rheumatology/keac054 35094047

[67] Heiblig M Patel BA Groarke EM Bourbon E Sujobert P. Toward a pathophysiology inspired treatment of VEXAS syndrome. Semin Hematol. (2021) 58:239–46. 10.1053/j.seminhematol.2021.09.001 34802546

[68] Fu Y Wu W Chen Z Gu L Wang X Ye S. Trisomy 8 Associated Clonal Cytopenia Featured With Acquired Auto-Inflammation and Its Response to JAK Inhibitors. Front Med. (2022) 9:895965. 10.3389/fmed.2022.895965 35547205 PMC9082665

[69] Heiblig M Ferrada MA Koster MJ Barba T Gerfaud-Valentin M Mékinian A. Ruxolitinib is more effective than other JAK inhibitors to treat VEXAS syndrome: a retrospective multi-center study. Blood. (2022). 10.1182/blood.2022016642 35609174 PMC9412002

[70] Watanabe R Hashimoto M. Perspectives of JAK Inhibitors for large vessel vasculitis. Front Immunol. (2022) 13:881705. 10.3389/fimmu.2022.881705 35432355 PMC9005632

[71] Silverman LR Demakos EP Peterson BL Kornblith AB Holland JC Odchimar-Reissig R. Randomized controlled trial of azacitidine in patients with the myelodysplastic syndrome: a study of the cancer and leukemia group B. J Clin Oncol. (2002) 20:2429–40. 10.1200/JCO.2002.04.117 12011120

[72] Cordts I Hecker JS Gauck D Park J Härtl J Günthner R. Successful treatment with azacitidine in VEXAS syndrome with prominent myofasciitis. Rheumatology. (2022) 61:e117–9. 10.1093/rheumatology/keab866 34894213

[73] Comont T Heiblig M Rivière E Terriou L Rossignol J Bouscary D. Azacitidine for patients with vacuoles, E1 enzyme, X-linked, autoinflammatory, somatic syndrome (VEXAS) and myelodysplastic syndrome: data from the French VEXAS registry. Br J Haematol. (2022) 196:969–74. 10.1111/bjh.17893 34651299

[74] Loschi M Roux C Sudaka I Ferrero-Vacher C Marceau-Renaut A Duployez N. Allogeneic stem cell transplantation as a curative therapeutic approach for VEXAS syndrome: a case report. Bone Marrow Transplant. (2022) 57:315–8. 10.1038/s41409-021-01544-y 34999727

[75] Claussnitzer M Cho JH Collins R Cox NJ Dermitzakis ET Hurles ME. A brief history of human disease genetics. Nature. (2020) 577:179–89. 10.1038/s41586-019-1879-7 31915397 PMC7405896

[76] Mahmud S Biswas S Afrose S Mita MA Hasan MR Shimu MSS. Use of next-generation sequencing for identifying mitochondrial disorders. Curr Issues Mol Biol. (2022) 44:1127–48. 10.3390/cimb44030074 35723297 PMC8947152

[77] Levy-Lahad E King MC. Hiding in plain sight - somatic mutation in human disease. N Engl J Med. (2020) 383:2680–2. 10.1056/NEJMe2030754 33108100

